# Extremely Stretchable Strain Sensors Based on Conductive Self‐Healing Dynamic Cross‐Links Hydrogels for Human‐Motion Detection

**DOI:** 10.1002/advs.201600190

**Published:** 2016-09-07

**Authors:** Guofa Cai, Jiangxin Wang, Kai Qian, Jingwei Chen, Shaohui Li, Pooi See Lee

**Affiliations:** ^1^ School of Materials Science and EngineeringNanyang Technological University50 Nanyang AvenueSingapore639798

**Keywords:** human‐motion detection, hydrogels, self‐healing, soft devices, stretchable strain sensors

## Abstract

**Extremely stretchable self‐healing strain sensors based on conductive hydrogels** are successfully fabricated. The strain sensor can achieve autonomic self‐heal electrically and mechanically under ambient conditions, and can sustain extreme elastic strain (1000%) with high gauge factor of 1.51. Furthermore, the strain sensors have good response, signal stability, and repeatability under various human motion detections.

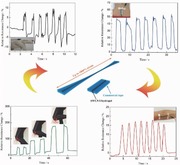

Stretchable, wearable, flexible, and human friendly soft electronic devices are of significance to meet the escalating requirements of increasing complexity and multifunctionality of modern electronics.^1^, ^2^, ^3^, ^4^, ^5^, ^6^ Strain sensors can generate repeatable electrical changes upon mechanical deformations. They have found particular interest and broad applications in robotics, sports, health monitor, and therapeutics, etc. To date, several representative strain sensors using carbon nanotubes,^7^, ^8^, ^9^ metal/semiconductor,^10^, ^11^, ^12^ graphene,^13^, ^14^, ^15^ conductive polymer,^16^, ^17^ and microfluidic^18^, ^19^ as conductive materials combining with elastomeric substrates have been successfully fabricated. However, most of these devices can only be stretched to a very limited extent (usually less than 200%). Lewis and co‐workers^20^ have developed a capacitive soft strain sensor using an ionically conductive fluid and silicone elastomer as the conductor and dielectric/encapsulant respectively, which can be stretched up to 700%, but the gauge factor is small (0.348 ± 0.11). We can define the gauge factor as (Δ*R*/*R*_0_)/*ε*, where Δ*R*/*R*_0_ is relative resistance change, *R*_0_ is the resistance at 0% strain, *R* is the resistance under stretch, and *ε* is the applied strain.^21^ In addition, introducing self‐healing properties to these soft electronic devices that can repeatably recover mechanical and electrical performance under room temperature, even at the same damaged location or under extremely stretchable situation, is of high importance to avoid the degradation of the device performance during the deformation.

Nowadays, self‐healing materials have attracted increasing attention, especially in soft electronics field. Haick and co‐workers^22^ have reported a self‐healing flexible sensing platform by dispersing metal particles in polyurethane diol as self‐healing electrode. Bao and co‐workers^23^ have demonstrated a self‐healing electronic sensor skin based on nanostructured μNi particles‐supramolecular organic composite. Park and co‐workers^24^ have developed self‐healing conductive hydrogel by polymerizing pyrrole in agarose solution. However, none of these self‐healing electronic devices can be stretched over 100%. Recently, there are intense research on highly stretchable hydrogels, which are mainly focusing on ionic conductors due to their excellent transparency and small resistance variation under high stretching states.^25^, ^26^, ^27^, ^28^, ^29^ In particular, conductive hydrogels are promising materials for the fabrication of ionic skin, bioelectrodes, and biosensors because many hydrogels with high water concentration have biocompatibility properties.^24^, ^30^, ^31^, ^32^ Therefore, it is of great interest to fabricate highly stretchable self‐healing strain sensor by combining the advantages of both biocompatible hydrogels and electronic conductors for applications in robotics, human motion detection, entertainment, medical monitoring, and treatment etc.

Herein, we introduce a new type of extremely stretchable self‐healing piezoresistive strain sensor using different electronic conductors comprised of single wall carbon nanotube (SWCNT), graphene, and silver nanowire in self‐healing hydrogel (SWCNT, graphene, and silver nanowire/hydrogel) as the conductive sensing channel built on a commercial transparent elastic substrate. The conductive hydrogel exhibits a fast self‐healing capability which can restore 98 ± 0.8% of its initial conductivity within 3.2 s healing time. Moreover, no external stimuli (such as heat, pH, light, or catalyst) are required. The fast self‐healing process of the SWCNT/hydrogel ensures rapid recovery of the electrical property of the sensor after being released to the relaxed state and avoids the degradation of the device performance during the large deformation. The self‐healing strain sensor is capable of monitoring strain, flexion, and twist forces. Moreover, it can measure and withstand strain up to 1000%, with high gauge factor and excellent cycling stability. Based on these key features, the self‐healing strain sensor can be used to accurately detect large‐scale human motion by embedding it in gloves, garments, or directly attaching it on skin. The present methodology developed paves the way for practical applications of highly stretchable self‐healing strain electronic devices.

The fabrication process of conductive hydrogel is illustrated in **Figure** **1**a (see the Experimental Section in the Supporting Information for details). Figure 1b illustrates the key reaction in forming crosslinked hydrogel. Borax, the salt of a strong base and a weak acid, is hydrolyzed in aqueous solution, yielding a boric acid/tetrafunctional borate ion. In the gelation experiments, trigonal planar B(OH)_3_ and tetrahedral B(OH)_4_^−^ exist as monomeric species due to the low concentration of borax employed (0.02 m). B(OH)_3_ is capable of complexing polyvinyl alcohol (PVA), however, it cannot produce polyol gels. The main reason is that the complexation reaction occurs through the attachment of boron to adjacent alcohol groups of the same polymeric chain and this prevents cross‐linking from taking place. Therefore, the crosslinked hydrogel is formed via tetrafunctional borate ion interaction with –OH group of PVA. The process is particularly effective in forming 3D gel networks. The hydrogen‐bonding between tetrafunctional borate ion and –OH group provides the self‐healing function because the cross‐link is so weak that it is neither resemblance of covalent bond character nor esterification involved.^33^, ^34^, ^35^ The hydrogen‐bonding can be easily broken and reformed, allowing the hydrogel to self‐heal and reform. The cross‐links are dynamically associated and dissociated readily under room temperature. The network cross‐linked by weak hydrogen‐bonding is easily disrupted by a mechanical deformation, however, it is relatively facile for the bonds to reform due to proximity of plenty –OH groups and borate ions, hence allowing self‐healing at room temperature. In addition, the PVA‐borax hydrogel exhibits non‐Newtonian behavior, resulting in flow under low stress and limited dimensional stability.^35^, ^36^ Therefore, the sufficient mobility of polymer chain and free tetrafunctional borate ions enables the hydrogen bond across broken interfaces to trigger the self‐healing process rapidly and without the need of external stimuli. Although the PVA itself can form hydrogel and autonomously self‐healing property according to the previous reports, the concentration of the PVA used is very high and the stretchability is limited.^37^, ^38^, ^39^ Before forming the hydrogel, SWCNT and 4 wt% PVA solution were homogeneously mixed under the surfactant assistance of BYK 348 which is a polyether modified siloxane (purchased from BYK‐Chemie GmbH). The SWCNT and water are wrapped in the 3D networks during the hydrogel crosslinking process, thereby the conductive self‐healing hydrogel was formed as shown in Figure 1c. It is worth noting that most of the free volume (or pores) within the hydrogel is taken up by water. The hydrogel is composed of water with weight percentage more than 95 wt%. The scanning electron microscope (SEM) micrographs taken from freeze‐dried SWCNT/hydrogel are shown in Figure 1d,e. The microstructure of the freeze‐dried SWCNT/hydrogel is 3D porous networks cross‐linked by the SWCNT and some immobilized polymer. The porous structure of the SWCNT and polymeric network inside the SWCNT/hydrogel is highly beneficial to the stretchability and facilitating rapid response of hydrogels. In order to reveal the interactions between the PVA and tetrafunctional borate ion, Fourier transform infrared spectroscopy (FTIR) experiment was conducted on Spectrum GX FTIR Spectrometer. As shown in Figure S1 (Supporting Information), the broad and strong peak around 3400 cm^−1^ is attributed to the symmetrical stretching vibration of –OH groups. The –OH stretching peak is sensitive to hydrogen bonding. Compared with pure PVA, the –OH stretching peak shifts to a higher wavenumber and the peak is enhanced after formation of the hydrogel, indicating the presence of hydrogen bonding interactions between the hydroxyl groups on the PVA molecular chains and tetrafunctional borate ion.^40^, ^41^ In addition, dynamic mechanical measurements of the pure hydrogel and SWCNT/hydrogel were carried out to investigate their rheological properties. Figure S2 (Supporting Information) shows the changes in the storage (G′, solid symbols) and loss modulus (G″, hollow symbols) as a function of angular frequency for hydrogel and SWCNT/hydrogel. It can be seen that the presence of SWCNT raises the moduli and enhances the elastic response of the hydrogel. In addition, both hydrogels have a solid behavior with the storage modulus exceeding the loss modulus over the entire frequency range.

**Figure 1:**
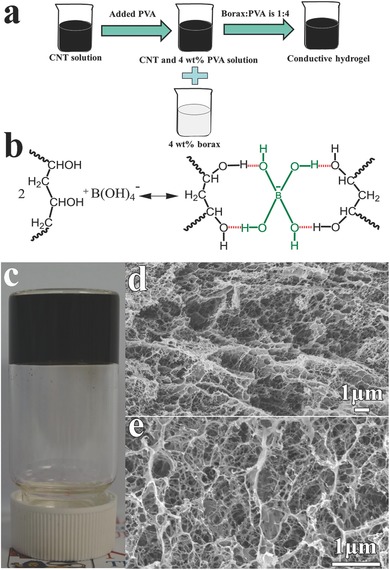
a) The fabrication process of conductive hydrogel. b) Crosslinking reaction between PVA and tetrafunctional borate ion. c) Photo image of SWCNT/hydrogel. d,e) SEM images of the freeze‐dried SWCNT/hydrogel.

**Figure** **2**a_1_–a_3_ shows the representative optical microscope images of how the SWCNT/hydrogel was healed after being completely separated by a scapel. The two fractured surfaces rapidly contact each other after the scapel was removed. The cutting groove was partially healed after 30 s and totally restored to normal after 60 s at room temperature without any external assistance (such as heat, light, and force). To further investigate the healing property of the SWCNT/hydrogel and recovery of the conductivity, the SWCNT/hydrogel was completely bifurcated and then the two furcated parts were rapidly brought together. Figure 2b presents the resistance changes over time of the SWCNT/hydrogel during the cutting and healing process. Once the conductive hydrogel was completely cut off, an open circuit was formed and the resistance changed to infinity. As the two furcated parts were brought together, the resistance dropped quickly and the resistance reached a constant value within 3.2 s. In addition, the self‐healing efficiency of the SWCNT/hydrogel was calculated by Rr/Ri (Rr is the recovered conductivity and Ri is the initial conductivity). Rr/Ri is 98.6% after healing for 3.2 s. It is worth noting that the resistance is lower than that of original value at the moment the two furcated parts get in contact, which is due to the transfer of free ions in the hydrogel, similar phenomenon was observed in the reduced graphene oxide based hydrogel.^42^ Figure 2c shows the repetitive cutting‐healing processes with five cycles at the same location. The resistance of the sample is relatively stable during the cycling. The high self‐healing efficiency was observed in each cutting‐healing process (Figure S3, Supporting Information). The average efficiencies are 98 ± 0.8% for the five self‐healing cycles within about 3.2 s, indicating the SWCNT/hydrogel possesses significant and repeatable electrical restoration performance.

**Figure 2:**
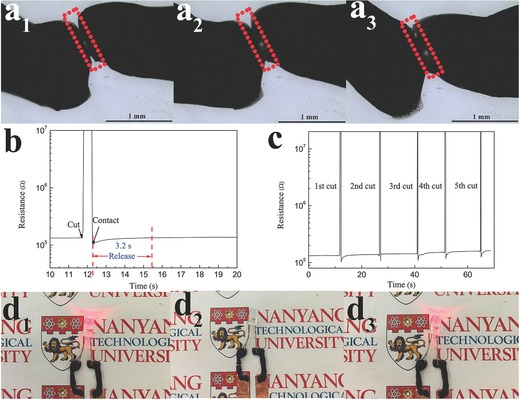
a) In situ self‐healing optical images of the SWCNT/hydrogel at room temperature, the healing time of (a_1_–a_3_) are 0, 30, 60 s, respectively. b) Time evolution of the electrical healing process by resistance measurements under ambient conditions. c) cycling of the cutting‐healing processes at the same location. d) Circuit comprises self‐healing SWCNT/hydrogel in series with an LED indicator, d_1_) undamaged, d_2_) completely bifurcated, and d_3_) electrical healing.

The self‐healing property of SWCNT/hydrogel was also demonstrated on a complete circuit composed of a LED indicator with SWCNT/hydrogel as the conductor, as shown in Figure 2d_1_–d_3_. The LED indicator was successfully lighted when a driving voltage of 5 V was applied. The LED indicator was extinguished when the SWCNT/hydrogel was completely bifurcated and the circuit became open‐circuit state. Once the two furcated parts were partially brought together, the circuit was restored and the LED indicator could be lighted up again. The demonstration here illustrates that the SWCNT/hydrogel has great potential in applications of self‐healing electronic device such as biosensors, electronic skin, wearable electronics, and so on.

To evaluate the performance of the strain sensor, we realized the self‐healing piezoresistive strain sensor using the SWCNT/hydrogel as conductor, and Scotch permanent clear mounting tape (VHB 4010, 3 m) as elastomeric substrates and encapsulant, as shown in **Figure** **3**a. The high stretchability of both SWCNT/hydrogel and VHB tape allowed the self‐healing strain sensor to remain intact up to 1000% strain, the highest value for electronic strain sensor so far, to the best of our knowledge.^43^, ^44^, ^45^ The excellent performances of the device are derived from all parts of the device or their coordination with each other. Although the SWCNT/hydrogel itself could not be recovered to the initial state under extreme strain conditions, it can work well when attached to the VHB tape. A strain sensor using the hydrogel without the electronic conducting component (SWCNT) was also prepared on the elastic substrate with the same parameters for comparison. Relative resistance changes versus strains are shown in Figure 3b. The relative resistance change increases with increasing tensile strain. A relative resistance change [(*R* −*R*_0_)/*R*_0_ = Δ*R*/*R*_0_, *R*_0_ is the resistance at 0% strain, *R* is the resistance under stretch] of 1514% was observed at 1000% strain for SWCNT/hydrogel, the sensitivity is nearly three times higher than that of the hydrogel without electronic conductor (533%). The large resistance change is highly desired for strain sensing applications, which is prerequisite for high sensitivity. Moreover, the SWCNT/hydrogel based strain sensor also showed reproducible and reliable responses to the small strain from 2% to 100% (Figure S4, Supporting Information). These results indicate that the SWCNT/hydrogel based strain sensor can work well from small strain to extreme strain. There are two aspects leading to the piezoresistive effects of SWCNT/hydrogel: one is the intrinsic piezoresistivity of the hydrogel, the other is the change of the contact conditions of SWCNT for electron conduction, such as contact area, loss of contacts and spacing variations upon stretching, and so on. The electrical conductivity of hydrogel without electronic conductor comes from ions conductivity (such as Na^+^, H^+^ in the hydrogel).^26^, ^32^ In addition, the relative resistance changes versus strains of the strain sensor can fit into a parabolic equation y = A*ε* ^2^ + B*ε* + C, where y is the relative resistance changes and *ε* is the tensile strain.^23^, ^46^ There is no relative resistance change when there is no strain applied to the sensor, so C is zero in the equation. Moreover, the value A can be defined as the sensitivity factor. Larger A value leads to more relative resistance changes, corresponding to higher sensitivity in the sensor. Hence, the sensitivity of the strain sensor can be quantified by the equation in Figure 3b. It can be seen that the SWCNT/hydrogel based strain sensor possesses a larger A than that of the hydrogel without electronic conductor. Therefore, the sensitivity of the SWCNT/hydrogel based strain sensor is higher than that of the hydrogel without electronic conductor, which is consistent with the experimental data. Figure S5 (Supporting Information) shows the photographs of the SWCNT/hydrogel based self‐healing strain sensor stretched to different strains.

**Figure 3:**
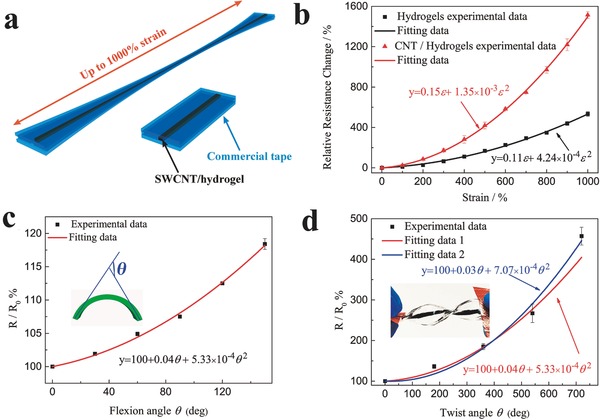
a) A piezoresistive strain sensor was fabricated by sandwiching a layer of conductive hydrogel between two layers of commercial tape (VHB 4010, 3 m)), which were then connected to two metallic electrodes, the piezoresistive strain sensor exhibited high extensibility up to 1000%. b) Plot of relative resistance change versus strain for SWCNT/hydrogel and hydrogel without electronic‐conductor‐based strain sensors. The equation represents a parabolic equation y = A*ε* ^2^ + B*ε* + C, where y is the relative resistance changes and *ε* is the tensile strain. c) Variation of normalized resistances as a function of flexion angle from 0° to 150°, inset presents the definition of flexion angle with bending radius of 2 cm. d) Variation of normalized resistances as a function of twist angle up to two revolutions. Equations in (c) and (d) represent a parabolic equation y = A*θ* ^2^ + B*θ* + C, where y is the resistance changes and *θ* is the flexion or twist angle. Error bars indicate standard deviation based on measurements of three devices.

Gauge factor represents the sensitivity of the sensors. Usually, brittle or poorly stretchable conductive materials have higher gauge factor. However, these materials do not possess or only sustain small stretchability. A relatively small strain could result in an irreversible fracture and lead to an infinite gauge factor. In the cases that high stretchability is required, the gauge factor of SWCNT/hydrogel was 0.24 at 100% strain and increased to 1.51 at 1000% strain (Figure S6, Supporting Information). The gauge factor is higher than that of the hydrogel without electronic conductor (from 0.09 at 100% strain to 0.53 at 1000% strain), and other piezoresistive electronic strain sensor (0.06 at 200% strain)^8^ and capacitive soft strain sensors based on ionic conductor (0.348 ± 0.11 at 700% strain).^20^ Although some strain sensors exhibit much higher gauge factors, the poor stretchability and lack of self‐healing capability restrict their applications under rigorous mechanical deformations.^47^, ^48^, ^49^

The responses of the SWCNT/hydrogel based strain sensor for other types of deformation such as flexion and twist which are related to human body movements were also tested. Figure 3c displays the resistance change as a function of flexion angle for the SWCNT/hydrogel based strain sensor. When the sensor was flexed, tensile stress built up at the outer curvature and compressive force built at the inner curvature. The conductive SWCNTs are separated from one another at the outer curvature and approached closer to one another at the inner curvature. However, the separated SWCNT played a dominant role for the device, thereby the resistance increased with increasing flexion angle. It can be seen that the resistance increased to 118% from its original value with bending angle increasing from 0° to 150°. When the strain sensor was twisted, the resistance changes versus twist angle still obeys the flexion parabolic equation within the twist angle of less than 540° as shown in the Figure 3d. However, the conductive SWCNT will be separated from one another around the twist point under larger twist angle (more than 540°), hence, the resistance of the device rapidly increases with increasing twist angle that detaches the SWCNT contacts or entanglements. The sensor responds to the twist angle with a good sensitivity, the resistance increases to 457% from the value of the untwisted state after two revolutions (720°) as shown in Figure 3d. The stability of the sensor was also investigated by repeatedly applying 700% stretching strain to the sensor and the resistance was measured at the released state (Figure S7, Supporting Information). The resistance of the sensor remains almost constant with minor fluctuations within 10% within the first 700 cycles strain test (between 0% to 700% strain). However, the largest resistance fluctuations were observed after 700 cycles due to partial water evaporation of the hydrogel during the long‐term cycles. The loss of water from hydrogels might become significant in long‐term cycles, which can be reduced by an appropriate encapsulation. Finding ideal packaging materials and technology for long lifetimes is a large undertaking beyond the scope of this paper. We suggest that SWCNT/hydrogel with 1000% stretchability is a superior candidate for strain sensing applications, considering the fast self‐healing property and significantly improved piezoresistive responses compared to the hydrogel without electronic conductor counterparts.

The excellent sensing performances were not only observed in SWCNT/hydrogel based strain sensor, but could also be achieved on graphene/hydrogel based strain sensor and silver nanowire/hydrogel based strain sensor (Figure S8 and Figure S9, Supporting Information). A relative resistance change of 916% and gauge factor of 0.92 were observed at 1000% strain for graphene/hydrogel. The silver nanowire/hydrogel based strain sensor exhibits high relative resistance change of 2249% and gauge factor of 2.25 at 1000% strain. However, the silver nanowire/hydrogel based strain sensor is not stable due to the easy oxidation of silver nanowires in water and air. The results indicate that the outstanding sensor performance can be obtained on various electronic conductor/hydrogel based strain sensors.

The self‐healing SWCNT/hydrogel is promising material platform for wearable strain sensor. For demonstration, the SWCNT/hydrogel based sensor was mounted on the white cotton glove, garment, or directly attached on the skin to detect the bending and stretching of the human body, such as finger knuckle, knee joint, neck and elbow joint. **Figure** **4**a shows the motion detection for the index finger. We checked the response behaviors of the SWCNT/hydrogel based strain sensor when the fingers were repeatedly bent at a frequency of 1 Hz. It can be seen that the sensor responded to the motion of the finger rapidly and repeatedly. The background noise is corresponding to the slight trembling of the finger that can be detected by the SWCNT/hydrogel strain sensor, indicating the high sensitivity of the sensor. Figure 4b illustrates the detection of knee joint bending, the sensor is stretched when the bending angles are at 79.8°, 92.5°, 122.7°, respectively, and released when straightening the knee, the resistance increases during the bending and recover during the releasing process. It can be seen that the resistance of the sensor increased as the bending angle increased. Moreover, the sensor is capable of distinguishing the different bending angles of the knee. When the knee was held at a certain angle, the resistance of SWCNT/hydrogel based strain sensor remained at a constant value and returned to the original value after straightening the knee. Furthermore, when the SWCNT/hydrogel based strain sensor was used to further monitor other human‐motion such as neck bending and elbow bending, sharp and rapid responses were also observed as shown in Figure 4c,d, respectively. Although a slight drift is noticed in these responses, the sensor exhibited good stability and repeatability in the signal. The hysteresis of the sensor in the response could lead to the signal drift. Figure S10 (Supporting Information) depicts the relative resistance changes of 1000% and 100% strain under stretching–releasing cycles, significant hysteresis is observed during the 1000% stretching–releasing cycling. The hysteresis is caused by rearrangement of the CNT in the hydrogel matrix and the considerable hysteresis of VHB tape. However, even in this case, the original resistance of the sensor is almost fully recovered after releasing it from strain and the hysteresis is negligible within 100% strain. Moreover, the sensor also has important advantages of low creep and fast response. As seen in Figure S11 (Supporting Information), a step strain of 100% was imposed within 1 s, revealing a percent overshoot and creep recovery time of 7.4% and 0.7 s, respectively, which can be attributed to the excellent self‐healing property of the conductive SWCNT/hydrogel. The differences in the degree of muscle movements could lead to a slight drift. In order to prove this, the response behavior of the sensor was also investigated by repeatedly applying 100% stretching strain to the sensor as shown in Figure S12 (Supporting Information). It can be seen that the sensor exhibits a negligible drift under the constant strain. These results demonstrate that our strain sensors can be used as human motion sensor and have the potential application in a wide range of stretchable devices.

**Figure 4:**
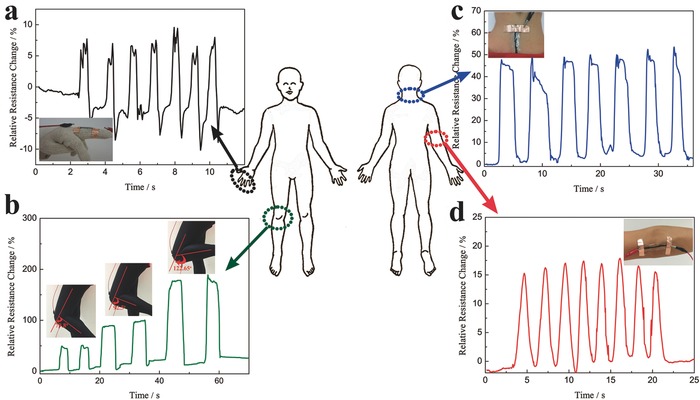
Monitoring various human motion in real time a) Relative resistance changes versus time for the bending and release of the index finger, the inset shows the sensor was fixed on a white cotton glove. b) Relative resistance changes versus time when bending the knee at different angles, the insets show the the sensor mounted on the knee at different bending angles at 79.8°, 92.5°, 122.7°, respectively. c) Relative resistance changes versus time for the neck bending and release, the inset presents the the sensor directly attached on the neck. d) Relative resistance changes versus time for the elbow bending and release, the inset presents the sensor directly attached on the elbow joint.

In summary, the present work first demonstrated the extremely stretchable self‐healing strain sensors based on various repeatable self‐healing conductive hydrogels such as SWCNT, graphene, and silver nanowire/hydrogel under ambient conditions. The conductive SWCNT/hydrogel exhibits fast electrical healing speed (within 3.2 s) and high self‐healing efficiency (98 ± 0.8%). The strain sensor is capable of sustaining severe elastic deformation (up to 1000%) with high gauge factor of 1.51. After being stretched to 700% strain for 1000 cycles, no significant change was observed in the intrinsic properties of the strain sensor. The electrical resistance can effectively recover by self‐healing of the conductive SWCNT/hydrogel. Furthermore, the strain sensor could effectively monitor and distinguish multifarious human motion when used as wearable strain sensor. We found that the SWCNT/hydrogel based strain sensors have good response, stability, and repeatability of the signal during the human motion detection measurements. We believe that our extremely stretchable self‐healing strain sensor could find a wide range of applications in robotics, sports, health monitoring, and medical treatments.

## Acknowledgements

This research is supported by the National Research Foundation Competitive Research Programme NRF‐CRP13‐2014‐02, and National Research Foundation Investigatorship Award NRF‐NRFI2016‐05 that is supported by the National Research Foundation, Prime Minister's Office, Singapore.

## Supporting information

As a service to our authors and readers, this journal provides supporting information supplied by the authors. Such materials are peer reviewed and may be re‐organized for online delivery, but are not copy‐edited or typeset. Technical support issues arising from supporting information (other than missing files) should be addressed to the authors.

Supplementary
