# Supplementary material for: Extremely Stretchable Strain Sensors Based on Conductive Self‐Healing Dynamic Cross‐Links Hydrogels for Human‐Motion Detection
Source: Adv Sci (Weinh). 2016 Sep 7;4(2):1600190. doi: 10.1002/advs.201600190 (PMC5323873; doi:10.1002/advs.201600190)
Supplement: Supplementary file 1 — Supplementary [file ADVS-4-na-s001.pdf]

Copyright WILEY-VCH Verlag GmbH & Co. KGaA, 69469 Weinheim, Germany, 2016.

ADVANCED SCIENCE
Open Access

Supporting Information

for *Adv. Sci.*, DOI: 10.1002/advs.201600190

Extremely Stretchable Strain Sensors Based on Conductive Self-Healing Dynamic Cross-Links Hydrogels for Human-Motion Detection

*Guofa Cai, Jiangxin Wang, Kai Qian, Jingwei Chen, Shaohui Li, and Pooi See Lee**

Supporting Information for

Extremely stretchable strain sensors based on conductive self-healing dynamic cross-links hydrogels for human-motion detection

*Guofa Cai, Jiangxin Wang, Kai Qian, Jingwei Chen, Shaohui Li, and Pooi See Lee**

[*] G. F. Cai, J. X. Wang, K. Qian, J. W. Chen, S. H. Li, Prof. P. S. Lee
School of Materials Science and Engineering
50 Nanyang Avenue
Nanyang Technological University
Singapore, 639798
E-mail: pslee@ntu.edu.sg

Experimental Section

Materials. SWCNT (P3-SWNT, carbonaceous purity >90%) was purchased from Carbon Solutions, Inc. Graphene (TNRGO, >99 wt%) was obtained from Time Nano. Silver nanowire (diameter 100 nm, length 200 μm) was purchased from XFNANO, Inc. Scotch permanent clear mounting tape (VHB 4010) was purchased from 3M. Sodium tetraborate (assay 99%) and poly (vinyl alcohol) (Mw 89000~98000, >99% hydrolyzed) were purchased from Sigma-Aldrich. Surfactant BYK348 was obtained from BYK-Chemie GmbH. All aqueous solutions were prepared from deionized water (Milli-Q, Millipore Corp). All the reagents were used as received without further purification.

Preparation of SWCNT/hydrogel. SWCNT was uniformly dispersed into 5 ml of deionized water containing 0.5 wt% BYK348 under sonication. Then, 0.2 g PVA was added to the above solution under stirring at 90 °C for 2 h. Meanwhile, 0.2 g sodium tetraborate was dissolved into 5 ml deionized water. Last, the borax aqueous solution and SWCNT/PVA solution were mixed in a 1:4 volumetric ratio by vigorous stirring, until a gel was obtained. The graphene/hydrogel and silver nanowire/hydrogel were prepared by a similar method as SWCNT/hydrogel. The concentration of the SWCNT, graphene and silver nanowire is the same (5 mg/ml). The hydrogel without electronic conductor was also prepared with the same parameters for comparison purpose.

Preparation of strain sensor. The strain sensor was assembled by employing the conductive hydrogel as conductor, VHB tape as elastomeric substrates and encapsulant. The top and bottom of the sensor were insulated using VHB to prevent evaporation of the hydrogel. Before the strain sensor was assembled, the surfaces of the hydrogels were dried with N_2 gas for 30 s to improve the adhesion between VHB and hydrogel by removing water from the hydrogel surfaces.

Characterization: The microstructures of the freeze-dried SWCNT/hydrogel were characterized using scanning electron microscopy (SEM, JEOL 7600F, Japan). The SWCNT/hydrogel was dried by freeze-drying (Scanvac CoolSafe 110-4 PRO 4lt freeze dryer) at -110 °C under 0.091 mbar prior to imaging. The FTIR experiment was conducted on Spectrum GX FTIR Spectrometer (PerkinElmer Inc). An optical microscope (Olympus BX51) was used to observe the in-situ self-healing process of SWCNT/hydrogel. Rheology experiments were conducted on an Anton Paar MCR 501 rheometer (Anton Paar) at room temperature. For the stretching tests, the sensors were fixed on home-built stretching stages to apply the different strains. For the flexion measurement, the sensor was attached on a PET substrate with a thickness

of 50 μm to accurately control the bending angle. For the human motion detection, the sensors were connected to the Keithley analyzer. All the relative resistance changes were measured using a Keithley analyzer (Model 4200).

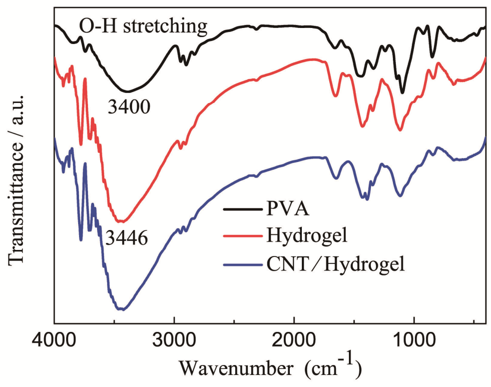

Figure S1 FTIR spectra of PVA, hydrogel and SWCNT/hydrogel.

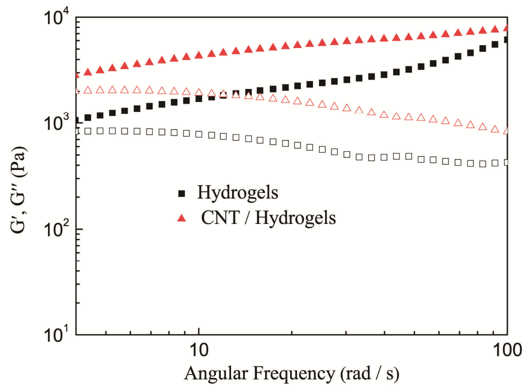

Figure S2 Storage modulus (G′, solid symbols) and loss modulus (G″, hollow symbols) as a function of dimensionless angular frequency for hydrogel and SWCNT/hydrogel.

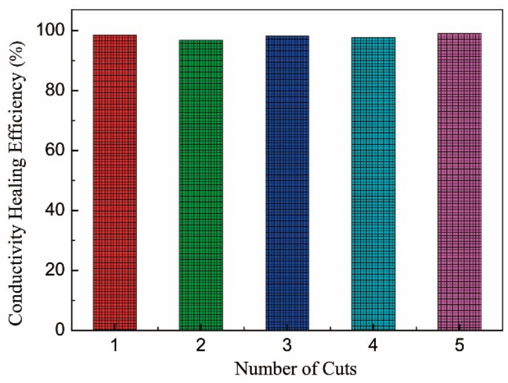

Figure S3 Conductive healing efficiencies as a function of the number of cuts.

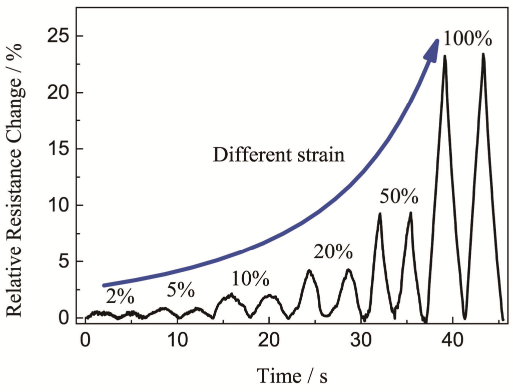

Figure S4 Plots of relative resistance change as the sensor was stretched from 2% to 100% strains for SWCNT/hydrogel.

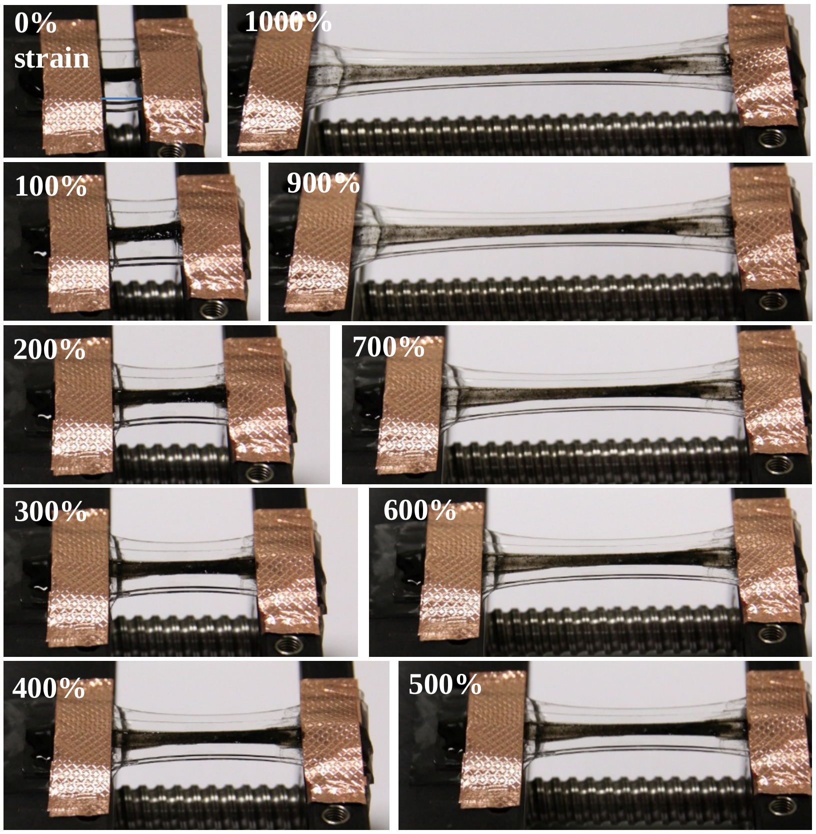

Figure S5 Photographs of the SWCNT/hydrogel based self-healing strain sensor stretched to different strains.

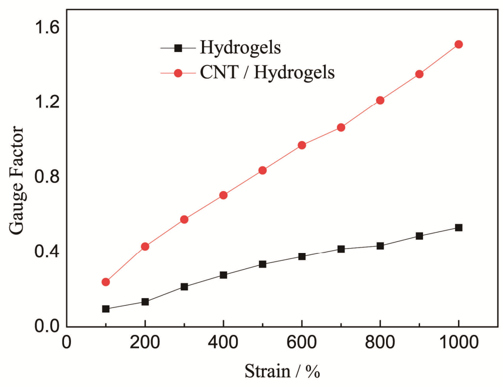

Figure S6 Gauge factors versus strain for SWCNT/hydrogel and hydrogel without SWCNT based strain sensors.

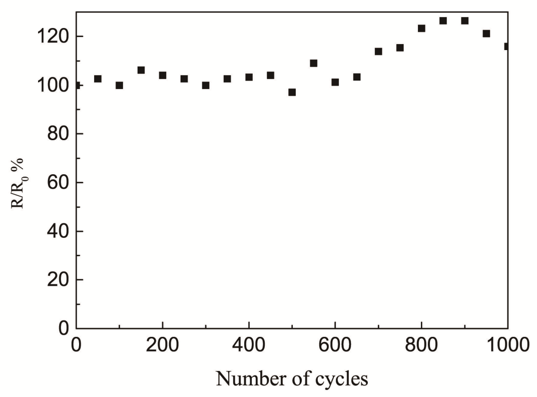

Figure S7 The stability of the SWCNT/hydrogel based strain sensor by repeatedly applying 700% strain for 1000 cycles.

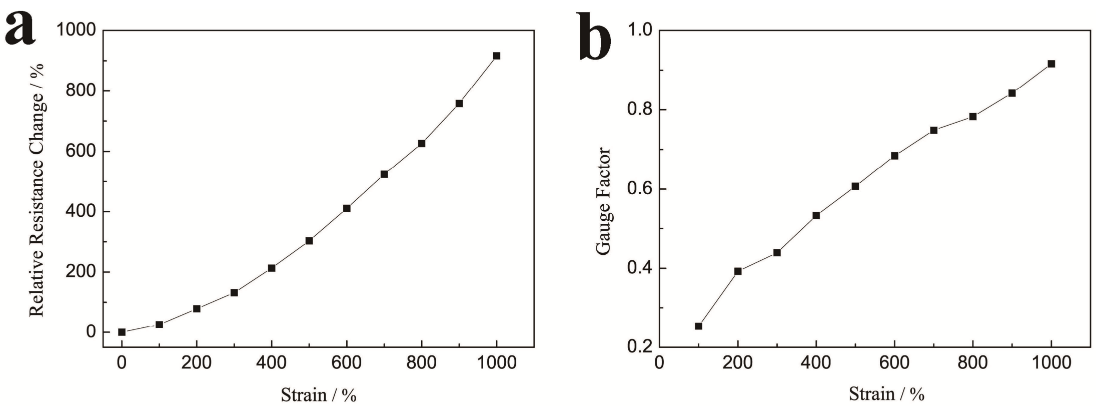

Figure S8 Plots of a) relative resistance change and b) gauge factors versus strain for graphene/hydrogel based strain sensor.

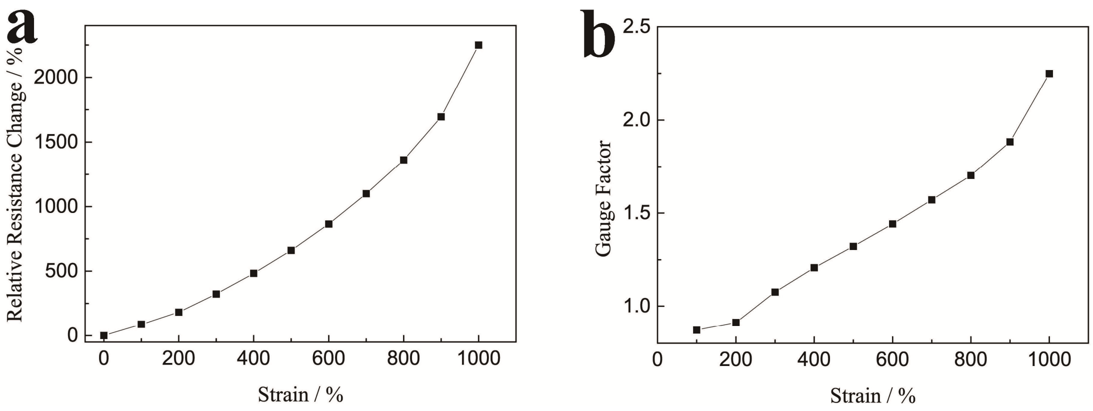

Figure S9 Plots of a) relative resistance change and b) gauge factors versus strain for silver nanowire/hydrogel based strain sensor.

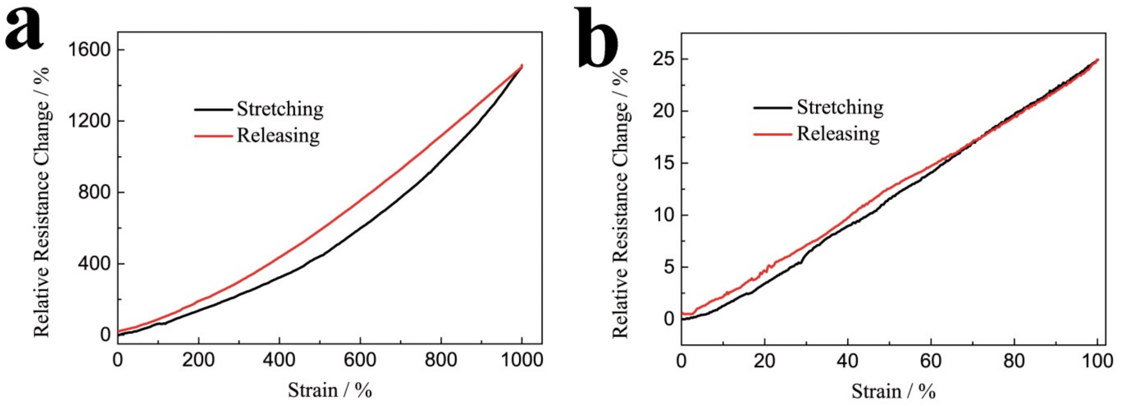

Figure S10 Relative resistance changes as a function of strain changes of a) 0%–1000%–0% and b) 0%–100%–0% with stage moving speed at 2 mm s^{-1}.

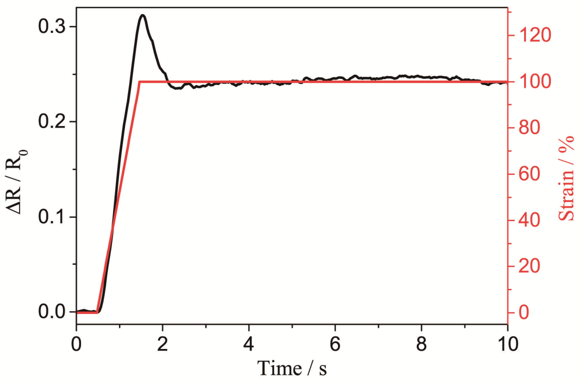

Figure S11 Creep recovery tests with step-strain of 100% are imposed in 1s.

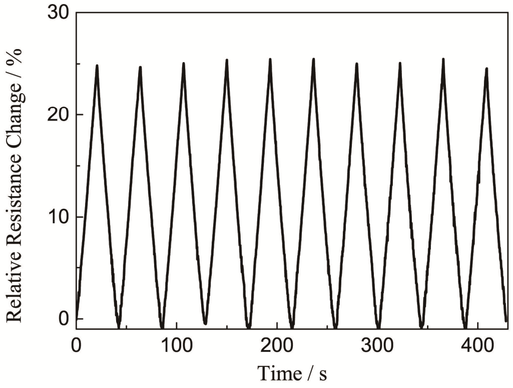

Figure S12 Response behavior of the sensor stretched from 0% to 100% strains.
